# Reduction in vertical transmission rate of bean common mosaic virus in bee-pollinated common bean plants

**DOI:** 10.1186/s12985-024-02407-w

**Published:** 2024-06-28

**Authors:** Netsai M. Mhlanga, Adrienne E. Pate, Warren Arinaitwe, John P. Carr, Alex M. Murphy

**Affiliations:** 1 National Institute of Agricultural Botany, New Rd, East Malling, West Malling, ME19 6BJ UK; 2 Department of Plant Sciences, University of Cambridge, Downing Street, Cambridge, CB2 3EA UK; 3 International Centre for Tropical Agriculture (CIAT), Dong Dok, Ban Nongviengkham, Vientiane, Lao People’s Democratic Republic

**Keywords:** *Phaseolus vulgaris*, Vertical transmission, Bean common mosaic virus, Bean common mosaic necrosis virus, Cucumber mosaic virus, Pollen, Pollination, Bumblebee

## Abstract

Vertical transmission, the transfer of pathogens across generations, is a critical mechanism for the persistence of plant viruses. The transmission mechanisms are diverse, involving direct invasion through the suspensor and virus entry into developing gametes before achieving symplastic isolation. Despite the progress in understanding vertical virus transmission, the environmental factors influencing this process remain largely unexplored. We investigated the complex interplay between vertical transmission of plant viruses and pollination dynamics, focusing on common bean (*Phaseolus vulgaris*). The intricate relationship between plants and pollinators, especially bees, is essential for global ecosystems and crop productivity. We explored the impact of virus infection on seed transmission rates, with a particular emphasis on bean common mosaic virus (BCMV), bean common mosaic necrosis virus (BCMNV), and cucumber mosaic virus (CMV). Under controlled growth conditions, BCMNV exhibited the highest seed transmission rate, followed by BCMV and CMV. Notably, in the field, bee-pollinated BCMV-infected plants showed a reduced transmission rate compared to self-pollinated plants. This highlights the influence of pollinators on virus transmission dynamics. The findings demonstrate the virus-specific nature of seed transmission and underscore the importance of considering environmental factors, such as pollination, in understanding and managing plant virus spread.

## Main text

We have previously shown that virus-infected plants exhibit the ability to influence their animal pollinators. For instance, infected tomato (*Solanum tuberosum* L.) and bean (*Phaseolus vulgaris* L.) plants emit volatile organic compounds that attract bumblebees (*Bombus terrestris* L.) [12, 21]. Furthermore, the negative impact of virus infection on tomato seed yield was ameliorated by bee pollination, and tomato pollen transfer by bumblebees was biased in favor of virus-infected plants in cross-pollination [12, 23], underscoring the complexity of these ecological interactions. In this study, we investigated the vertical transmission of the most important viruses limiting *Phaseolus vulgaris* (common bean) bean yield: the potyviruses bean common mosaic virus (BCMV) and bean common mosaic necrosis virus (BCMNV) and the cucumovirus, cucumber mosaic virus (CMV) [22, 31, 32, 35]. These viruses are primarily transmitted by aphids, with seed-borne transmission accounting for about 2% of the infections [5, 35]. Yet, despite the progress in understanding vertical virus transmission, the environmental factors exerting influence on this process remain largely unexplored [27]. We investigated vertical transmission of BCMNV, BCMV, and CMV in *P. vulgaris* plants that had been allowed to self-pollinate under controlled conditions, and also compared the seed transmission rate of BCMV in self-pollinated, hand-pollinated and bee-pollinated plants grown in a glasshouse and in an experimental field plot. By exploring both controlled and outdoor growth conditions, along with the influence of bee pollination, we aimed to unravel the environmental factors shaping the vertical transmission of these viruses. Our investigation not only contributes to the growing body of knowledge on plant virus ecology but also sheds light on the broader implications of these interactions for crop productivity and ecosystem dynamics.

Vertical transmission, the passage of pathogens from one generation to the next through seed or pollen, plays a pivotal role in the persistence of plant viruses. Vertical transmission ensures the sustained presence of viruses in plant populations across successive generations, even in the absence of suitable alternative hosts or vectors [26, 28]. In crops, infected seeds are a significant source of primary inoculum that can lead to damaging epidemics upon germination [13, 26]. Mechanisms governing vertical transmission are diverse, encompassing direct invasion through the suspensor and virus entry into developing gametes before symplastic isolation [28]. Seedborne viruses, associated with the maternally-derived seed coat, can also occasionally give rise to infected offspring, tobamoviruses being a notable example [6]. While vertical transmission garners increasing research attention, questions persist regarding the molecular mechanisms and environmental factors influencing this process [16, 27].

Notably, more than a quarter of known plant viruses exhibit vertical transmission [28, 30]. The intricate relationship between plants and their pollinators further complicates this dynamic. Animal-mediated pollination, particularly by bees, is indispensable for the reproduction of a substantial portion of major crops and an astounding 90% of wild flowering plants [1, 25]. Recent literature highlights the dual role of pollen not only in fertilisation but also as a potential vector for viruses infiltrating plant seeds [3, 10]. An increasing number of plant viruses have been identified within pollen [9, 10]. While the detection of a virus in pollen does not necessarily mean that a virus is pollen-transmitted, insect-assisted pollination nevertheless introduces a novel layer of complexity to the understanding of virus transmission dynamics.

## Differential rates of seed transmission for three bean viruses

**Fig. 1:**
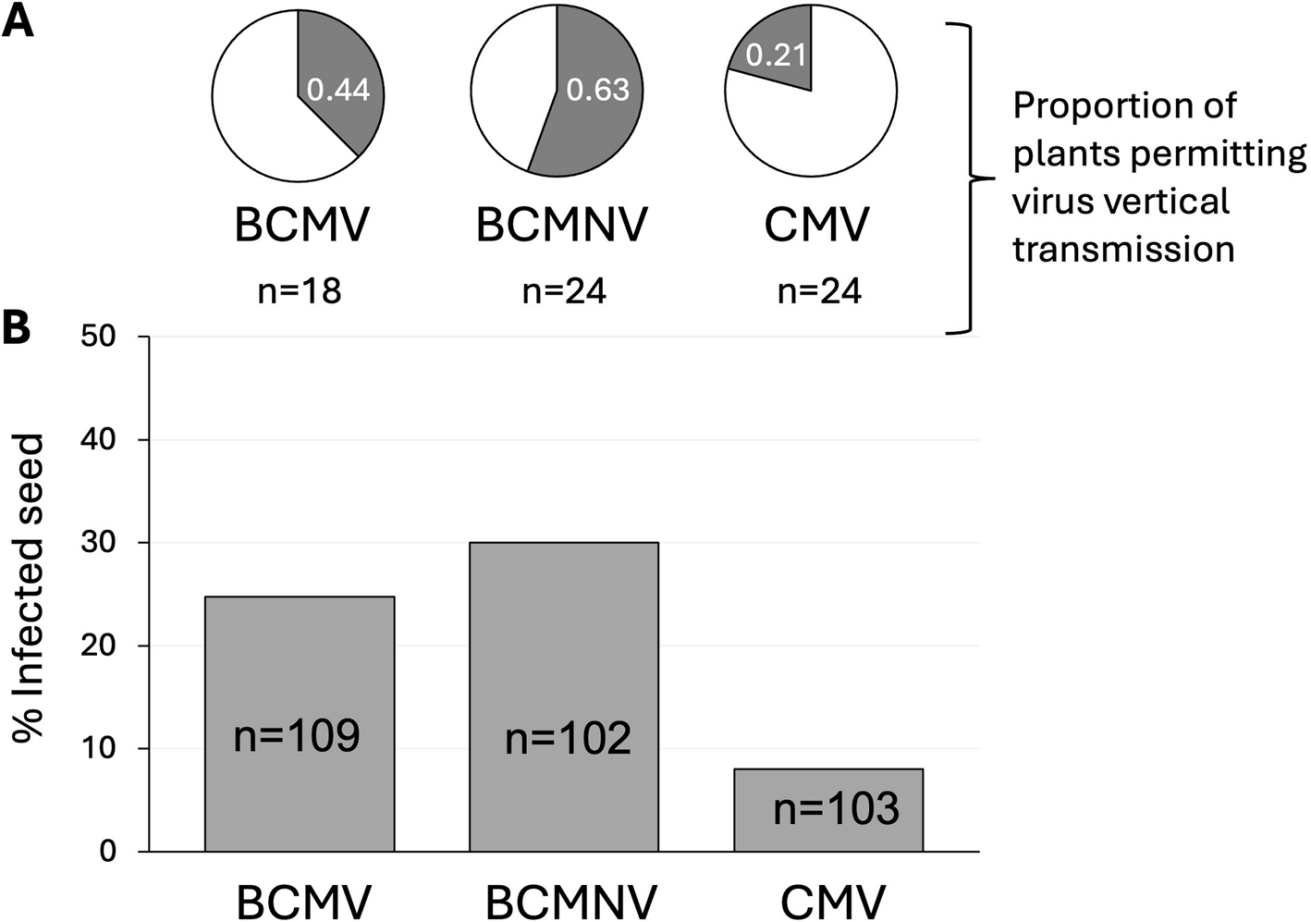
Rate of seed transmission for three viruses. Both at (**A**) parental and (**B**) progeny seedling levels, BCMNV recorded the highest seed transmission rate, followed by BCMV and CMV. Seeds from 18–24 plants in each treatment group were germinated for virus seed transmission testing. *n* = number of seedlings tested

Under controlled growth conditions (described in [33]), plants of *P. vulgaris* cv. ‘Wairimu’/Red Haricot-GLP 585 (Simlaw Seeds, Nairobi, Kenya) were mechanically inoculated with BCMNV (PV-0413), BCMV (PV-0915), or CMV (bean isolate PV-0473) (obtained from the German Collection of Microorganisms and Cell Cultures) 10 days post-germination on the first two true leaves. Seed progeny from virus-infected, self-pollinated bean plants from each treatment group were germinated and tested for the presence of virus using double-antibody sandwich enzyme-linked immunosorbent assay (ELISA) for BCMV, BCMNV, or CMV coat protein (Bioreba AG, Reinach, Switzerland). Vertical transmission of viruses from self-pollinated plants was highest for BCMNV (29.4%), followed by BCMV (22%) and CMV (8%) (Fig. 1B). The proportion of plants where seed transmission of the virus was detected was similarly highest for BCMNV-infected plants and lowest for CMV (Fig. 1A).

## Bee pollination under field conditions reduces the rate of BCMV seed transmission

**Fig. 2:**
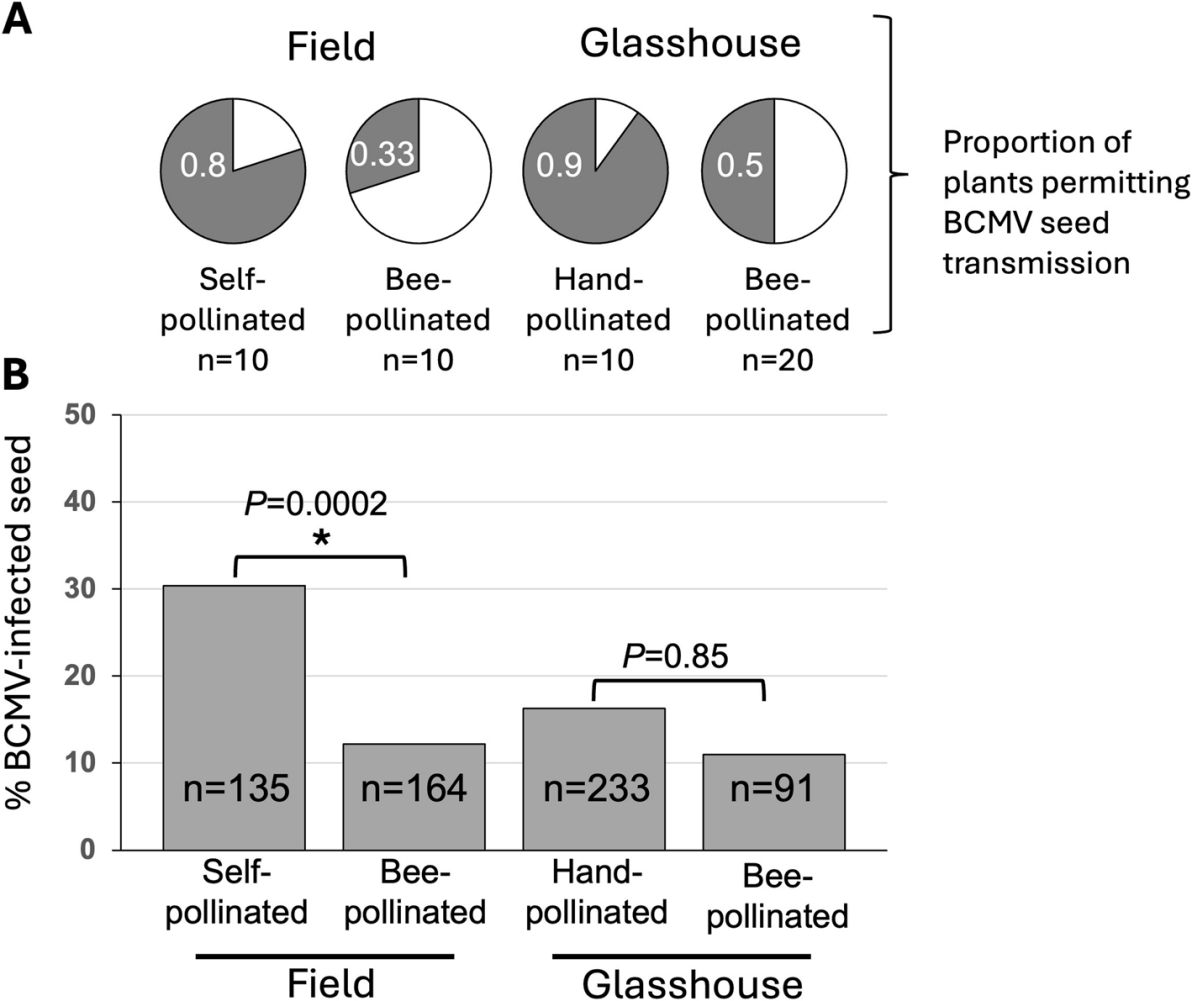
Bee pollination elicited a reduction in BCMV seed transmission both at parent plant and progeny seeding levels. Seed collected from BCMV-infected bean plants were germinated and tested for BCMV. The proportion of plants that permitted BCMV seed transmission (**A**) is shown. *n* = number of plants from which seed was collected. Plants grown in the greenhouse were either moved outdoors at the commencement of flowering and placed in the field plot so that wild bee pollinators could access the flowers or placed under bee-proof netting. In other experiments, flowering plants remained in the greenhouse and were either hand-pollinated by mechanically "tripping" the hull and wings of the bean flower to expose the stamens and pistil, or exposed to bumblebees that could pollinate freely. Seed harvested from bee- or hand-pollinated plants was germinated and tested for BCMV (**B**). There was a significant reduction in seed transmission of BCMV in outdoor bee-pollinated plants compared to plants under netting with no access to bees (Chi-square: *X*^2^ (1, *N* = 299) = 15.1, *p* = 0.000104). There was no significant difference in the seed transmission rate of hand- and bee-pollinated plants that remained in the greenhouse (Chi-square: *X*.^2^ (1, *N* = 324) = 0.127, *p* = 0.721)

**Fig. 3:**
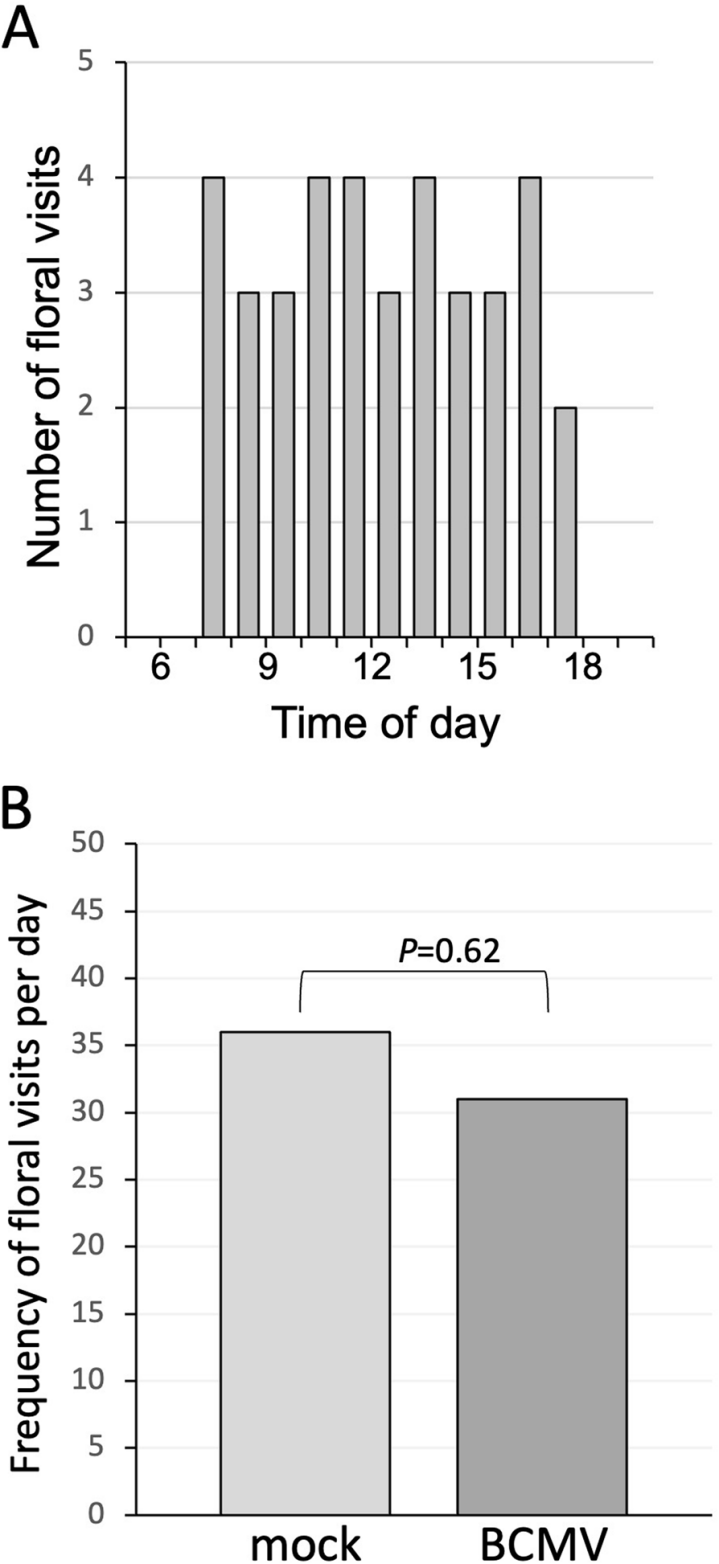
Bee visitation to uncovered flowering bean plants. An initial 14-h dawn to dusk observation indicated that only one species of bumblebee, *B. pascuorum* (common carder bee), visited an array of 10 uninfected and 10 BCMV-infected bean plants placed outside and that there was no peak foraging period (**A**). Over the subsequent seven days between 10:00 am and 2:00 pm, the frequency of bee visits to flowers of both mock-inoculated and BCMV-infected plants was recorded (**B**). The cumulative frequency is shown (equating to not more than 8 visits per day to any individual plant). Floral visitation frequency was similar to flowers of both mock-inoculated plants and BCMV-infected plants

Systemically-infected plants that had been inoculated 10 days post germination with BCMV, were transferred from a glasshouse to a field site (Botanic Garden Experimental Plot, University of Cambridge) at the onset of flowering (twenty days later). Plants were either kept under netting to exclude large insect pollinators, or left uncovered. Plants under netting had a BCMV seed-transmission rate of 30% (Fig. 2 C), comparable to the seed transmission rate of BCMV in plants grown in a controlled growth room (Fig. 1B). Most (8 out of 10) of the plants transferred outdoors produced a portion of offspring that were positive for BCMV (Fig. 2A). Remarkably, pollination by bees of the uncovered plants elicited a significant reduction in BCMV seed transmission rate to 12%. Daily observations of the uncovered plants revealed that one species of wild bumblebee visited flowers on these plants, *Bombus pascuorum* (common carder bee: Fig. 3A and B). The reduction in the rate of BCMV vertical transmission was also reflected in the lower proportion of plants that gave rise to infected progeny (Fig. 2A, C).

## Bee and hand-pollination under glasshouse conditions reduces the rate of BCMV seed transmission

Experimental plants were raised in batches. Plants were mechanically inoculated with BCMV 10 days post-inoculation on the first two true leaves. During the second week of flowering, half of each batch of plants were exposed to bumblebees, while the other half underwent hand-pollination at the same time, all under glasshouse conditions. Hand-pollination involved manually depressing the hull petal of the bean flower, or “tripping”, to force the stamens and stigma into physical contact to thereby enhance self-pollination.

Pollination of infected plants by *B. terrestris* from commercially produced colonies (Koppert Biological Systems, Berkel en Rodenrijs, The Netherlands) resulted in a comparably low rate of vertical transmission of BCMV as plants pollinated outdoors by wild bumblebees (Fig. 2B). Similarly, hand-pollination of common bean flowers resulted in a 16% BCMV transmission rate, not significantly different from the vertical transmission rate in bee-pollinated plants (Fig. 2).

## Discussion

Virus seed transmission can be achieved directly (internally) through invasion of the developing ovule (before fertilisation) or the embryo (after ferilisation) and indirectly by infected male gametes [28]. In direct embryo invasion after fertilisation, Wang and Maule [34] showed that viruses invade the embryo via the suspensor, which functions as a conduit for nutrient flow to support the growth of the embryo. Both direct and indirect embryo invasion processes can operate simultaneously for certain viruses in specific hosts, as observed in barley stripe mosaic virus in barley [19] and BCMV in common bean [20, 29]. Importantly, we found that BCMV-infected plants, when pollinated by bees under glasshouse and field conditions, or hand-pollinated, produced progeny seedlings with significantly lower BCMV transmission rates compared to plants where pollination was not assisted.

Although common bean is self-fertile, pollination services from almost exclusively hymenopteran insects can significantly increase seed yield [8, 11]. This is likely due to the improved efficiency of pollen deposition onto the stigma that occurs when large pollinators (i.e. bees and certain wasps) with sufficient weight and strength mechanically "trip" the hull and wings of the bean flower as they forage on the pollen and nectar [2]. Delivery of pollen loads to the stigma that exceed the threshold necessary to fertilise all ovules will lead to competition among pollen grains, which may improve offspring quality and maternal fitness [14]. Given that infected pollen is known to contribute to BCMV vertical transmission [20, 29], it is possible that the reduction in BCMV vertical transmission by bee or hand-assisted pollination could be explained by pollen competition with BCMV-free pollen outcompeting pollen carrying the virus, reducing the likelihood of embryo invasion. Although there is no direct evidence that BCMV-infected pollen is less fit than virus-free pollen, pollen from BCMV-infected plants has been shown to result in shorter germ tubes than pollen from healthy bean plants [20].

Our investigation into the efficiency of seed transmission rates revealed considerable variation among different virus species, with CMV exhibiting the lowest transmission rate, followed by BCMV, and BCMNV displaying the highest. Other studies in Arabidopsis have shown that the efficiency of poty- and cucomovirus vertical transmission was dependent on both the virus species and accession/ecotype of Arabidopsis [4]. The virus/host interaction factors influencing the likelihood of seed transmission include virus virulence, speed of movement within the host, multiplication in reproductive tissues, survival of virus-infected gametes and embryos, and host defence responses [4, 7, 15, 26, 28, 34]. Our results align with these ideas as we found that BCMNV, which is more virulent than BCMV in common bean, also had the highest rate of vertical transmission. However, Kyrychenko et al. (2020) [18] reported that *P. vulgaris* cv. Chervona Shapochka transmitted the Ukrainian BCMV strain in 77% of the seeds produced by infected plants, indicating an unusually high vertical transmission rate for this BCMV isolate that may be due either to the low resistance of the cultivar to BCMV, or the high virulence of BCMV strains circulating in common bean in Ukraine.

Hamelin et al. [13] and Pagán [26] emphasise the risks associated with seed transmission, particularly in introducing diseases to new areas, for example through the seed trade or when growers produce their own seed. Even an initial low incidence of seed transmitted virus has been known to initiate epidemics [17]. This is especially important for self-fertile leguminous crops in sub-Saharan Africa and South Asia where farmer produced seed is popular, as an alternative to the formal seed sector because of affordability [24]. 

This study expands our understanding of virus seed transmission dynamics and highlights the importance of pollinator behaviour in mediating and mitigating disease spread. Here, we suggest bee-pollination as an environmentally friendly method capable of both enhancing seed production and lowering the rate of virus-seed transmission.

## Data Availability

No datasets were generated or analysed during the current study.
